# Treatment with tumor necrosis factor inhibitors during pregnancy and occurrence of severe infections among exposed infants in Germany

**DOI:** 10.1186/s12884-025-08590-0

**Published:** 2025-12-20

**Authors:** Christina Princk, Nadine Wentzell, Marlies Onken, Kathrin Thöne, Bianca Kollhorst, Christof Schaefer, Katarina Dathe, Ulrike Haug

**Affiliations:** 1 Department of Clinical Epidemiology, Leibniz Institute for Prevention Research and Epidemiology – BIPS, Bremen, Germany; 2 Institut für Klinische Pharmakologie und Toxikologie, Charité – Universitätsmedizin Berlin, corporate member of Freie Universität Berlin, Humboldt-Universität zu Berlin, and Berlin Institute of Health, Pharmakovigilanz- und Beratungszentrum für Embryonaltoxikologie, Berlin, Germany; 3 Techniker Krankenkasse, Hamburg, Germany; 4 Department of Biometry and Data Management, Leibniz Institute for Prevention Research and Epidemiology – BIPS, Bremen, Germany; 5 Faculty of Human and Health Sciences, University of Bremen, Bremen, Germany

**Keywords:** Tumor necrosis factor inhibitor, Pregnancy, Infant, Infections

## Abstract

**Background:**

Tumor necrosis factor inhibitors (TNFi) are often used in the treatment of autoimmune diseases. Their use during pregnancy, particularly in the 2nd half, has been a matter of debate due to a potential risk of severe infections in infants. We aimed to describe (i) the dispensation of TNFi before and during pregnancy and (ii) the occurrence of hospitalizations with infections among prenatally exposed infants.

**Methods:**

Using the GePaRD database (claims data covering 20% of the German population), we included pregnancies ending in a live birth between 2006 and 2018 in women aged 12–50 years with ≥ 1 dispensation of TNFi in the year before or during pregnancy. We assessed maternal exposure to TNFi and hospitalization with infections among infants in the first year of life.

**Results:**

Among 1,113 children included, 265 (23.8%) were exposed to TNFi after the 20th gestational week. Among these, the proportion of infants hospitalized due to an infection in the first year of life was 11.7% (95% confidence interval [CI]: 8.4%-16.1%). In 529 children (47.5%), TNFi were dispensed only in the 365 days before pregnancy. Of these, 11.3% (95% CI: 8.9%-14.3%) were hospitalized due to an infection.

**Conclusions:**

Our analyses showed that among children whose mothers used TNFi in the year before or during pregnancy, about one fourth was exposed to TNFi in the second half of pregnancy. Among these children, the proportion hospitalized with a severe infection in the first year of life was similar to that from mothers exposed only before pregnancy.

**Supplementary Information:**

The online version contains supplementary material available at 10.1186/s12884-025-08590-0.

## Introduction

Several chronic inflammatory diseases are considerably more common in women than in men [1]. Although they can occur throughout life, their onset is often before the age of 50 and can thus also affect women of childbearing age who may become pregnant, intentionally or unintentionally [2, 3]. Providing health care to these women is a risk management challenge in many ways, and the ongoing trend towards late motherhood in many countries further increases the relevance of this topic [4]. On the one hand, treatment of chronic inflammatory diseases during pregnancy is important as periods of active disease can have negative effects on the mother’s health and increase, for example, the risk of premature birth [5, 6]. On the other hand, drug exposure during pregnancy may be associated with uncertainties regarding potential harm to the unborn child. This also applies to the use of tumor necrosis factor alpha inhibitors (TNFi) during pregnancy, which are often used in the treatment of chronic inflammatory diseases.

In the light of increasing evidence from observational data, recommendations for the use of TNFi during pregnancy have changed during the past years. For example, in 2008, a panel of international experts still disagreed whether TNFi should be stopped as soon as pregnancy is recognized or may be continued throughout pregnancy [7]. In 2008, the statement concluded that TNFi may be continued during pregnancy when strongly indicated. In 2016, a task force of the European League Against Rheumatism (EULAR) supported the use of TNFi in the first half of pregnancy [8]. However, there was still uncertainty whether the immunosuppressive effects of TNFi may increase the risk of severe infections in infants, particularly when they were exposed during the 2nd half of pregnancy. Given that exposure depends on the placental transfer of the different drugs, the EULAR statement from 2016 concluded that infliximab and adalimumab may preferentially be stopped at week 20 of pregnancy, and etanercept at week 30–32 of pregnancy. For certolizumab, it was noted that the safety of use throughout pregnancy still needed to be confirmed by extended published reports [8]. In the recently published updated guideline, treatment with certolizumab is stated as possible throughout the entire pregnancy [9]. The American College of Rheumatology guideline published in 2020 recommends certolizumab in all trimesters of pregnancy, whereas use of infliximab, etanercept, adalimumab and golimumab should be limited to the first and second trimesters [10]. The British Society for Rheumatology considers certolizumab treatment with its minimal placental transfer to be compatible in all three trimesters, while infliximab, adalimumab, etanercept or golimumab may be continued throughout pregnancy when necessary to maintain maternal disease control [11].

For inflammatory bowel disease, the consensus paper of the European Crohn’s and Colitis Organization (ECCO) published in 2015 stated that the risk of infection with TNFi alone or in combination with immunomodulators is controversial [12]. Furthermore, it mentioned that the timing of the last TNFi dose should consider both maternal disease activity and placental transfer of the drug. When considered appropriate by the clinician and the patient, the TNFi should be discontinued around gestational week 24–26 [12]. In 2023, the ECCO paper was updated [13]. It now states that women with active disease just before or during pregnancy, or with disease that is difficult to control, should continue TNFi or non-TNFi biologics throughout the pregnancy. In addition, women in remission should continue TNFi treatment throughout the pregnancy as discontinuation prior to the third trimester might increase the risk of relapse and lead to unfavorable pregnancy outcomes [13]. In the meantime, several studies have been published most of which providing reassuring results regarding the risk of infections in the first year of life among children exposed to TNFi *in utero* [14–23] (further details on these studies are provided in the discussion). However, there are still unanswered questions. For this reason, we aimed to (i) describe the dispensation of TNFi before and during pregnancy in Germany to get an overview of the treatment behavior and (ii) based on these data, describe the occurrence of infections leading to hospitalization in the first year of life among prenatally exposed infants.

## Methods

### Data source

We conducted this study based on the German Pharmacoepidemiological Research Database (GePaRD) with claims data from four statutory health insurance providers in Germany. GePaRD currently contains data on approximately 25 million persons who have been insured with one of the included providers since 2004 or later. There is information on approximately 20% of the German population per data year. Approximately 90% of the general population in Germany are insured with a statutory health insurance provider and the German health care system is characterized by uniform access to all levels of care.

All geographical regions of Germany are represented in GePaRD [24]. It contains demographic data, information on the dispensation of reimbursable drugs prescribed by physicians in the outpatient setting and on outpatient and inpatient services and diagnoses. Diagnoses are coded based on the German Modification (*ICD-10-GM*) of the *International Statistical Classification of Diseases and Related Health Problems*, version 10; the German coding system for operations and procedures („*Operationen- und Prozedurenschlüssel*”, *OPS*) is applied to code diagnostic and surgical/medical procedures, and outpatient services are coded according to the „*Einheitlicher Bewertungsmaßstab”* (*EBM*) [25–27]. Drugs in GePaRD are coded based on the Anatomical Therapeutic Chemical (ATC) classification system. Treatment with TNFi was identified based on relevant ATC and OPS codes for adalimumab (ADA), certolizumab pegol (CTZ), etanercept (ETA), golimumab (GOL) and infliximab (IFX) (Table S1). We applied algorithms which we previously developed for research on drug utilization and safety during pregnancy based on GePaRD. These algorithms facilitate identification and classification of pregnancy outcomes [28, 29], estimation of the beginning of pregnancy [30, 31] and linkage of mothers with their offspring [32].

### Study population and study design

We included all pregnancies ending in a live birth between January 2006 and December 2018 among women aged 12 to 50 years at pregnancy onset and for which the mother’s and the child’s data could be linked. Further inclusion criteria were a continuous health insurance in the 365 days prior to pregnancy as well as during pregnancy and at least one dispensation of TNFi medication in this time period. The children born from these pregnancies were categorized according to the timing of TNFi dispensation before or during pregnancy and followed up until the end of continuous health insurance, the end of the study period (December 31, 2019), or death, whichever occurred first. The outcome of interest during the follow-up period was hospitalization due to a severe infection in the first year of life.

### Study variables

We assessed the mothers’ age at the beginning of pregnancy and records of any diagnoses coded in the year before pregnancy that could be relevant as potential indication of TNFi. We distinguished between codes for inflammatory bowel disease, for inflammatory rheumatic disease or for associated diseases of the rheumatic spectrum (Table S2). With respect to pregnancies, we determined the duration in gestational weeks, the pregnancy outcome and whether the child was born by caesarean section.

To describe exposure characteristics to TNFis, we distinguished between mothers (I) with a dispensation only in the year before pregnancy, (II) with at least one dispensation between the beginning of pregnancy and the 20th gestational week but no dispensation during the remaining pregnancy and (III) with at least one dispensation after the 20th gestational week. Additionally, we defined variables describing the exposure for each trimester as well as variables distinguishing between TNFi with high placental transfer (IFX, ADA and GOL) vs. low placental transfer (ETA and CTZ) [33–38]. Of note, the time point of the exposure was assigned based on the date of the dispensation of a TNFi, which is an injection typically providing the dose for several days or weeks.

To determine the occurrence of severe infections we considered the diagnosis codes used by Bröms et al. 2020 [14] (Table S3) and assessed whether they were recorded as hospital discharge diagnoses in the infants’ first year of life overall and separately for different kinds of infections.

### Data analysis

Based on the study variables (see above), we described the included pregnancies and the children using absolute numbers and proportions. For continuous variables, we calculated means with standard deviation and medians with interquartile ranges. For the proportions of infants hospitalized with an infection during the first year of life we calculated 95% confidence intervals. All statistical analyses were conducted using the software SAS version 9.4.

## Results

**Fig. 1:**
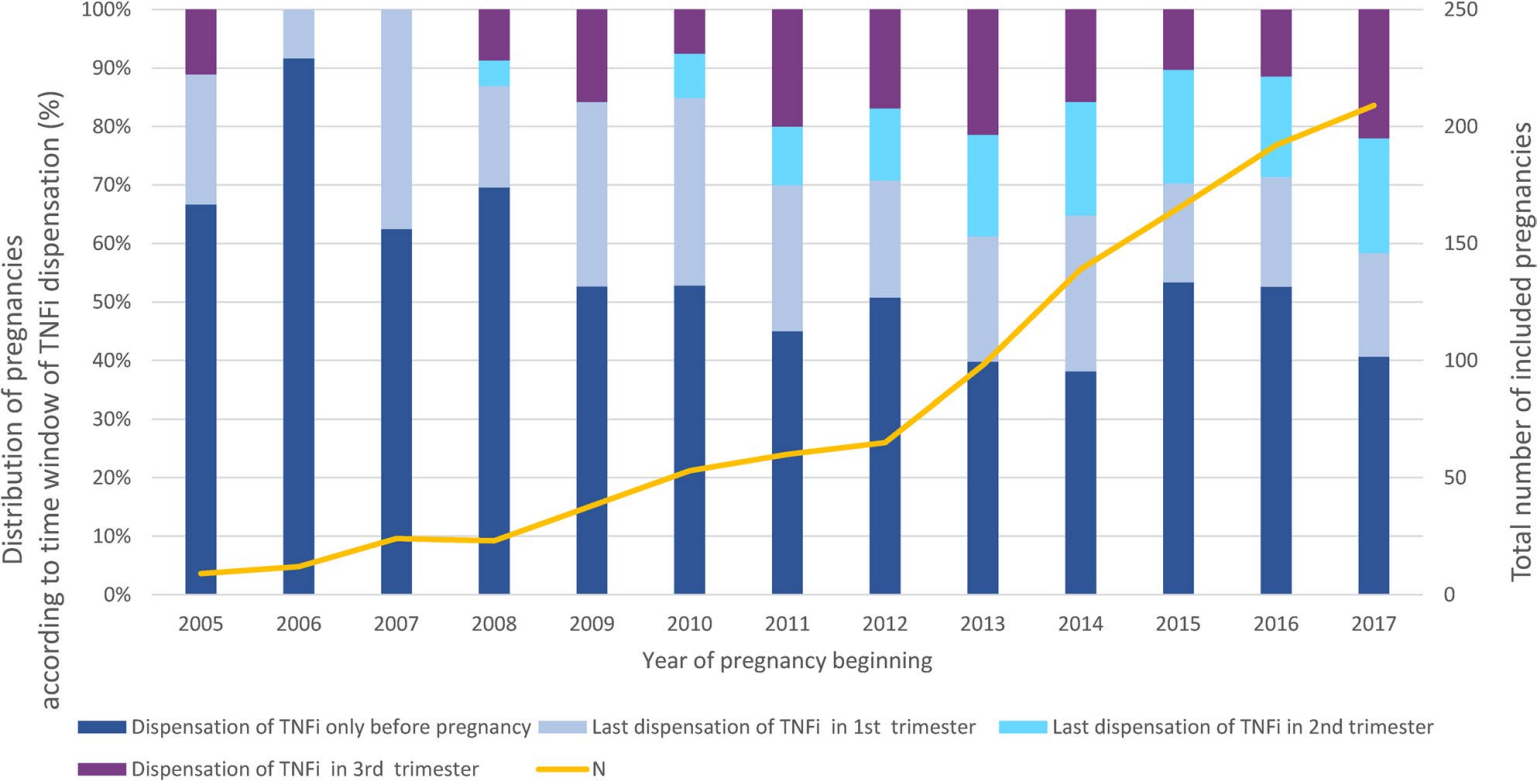
Total number of included pregnancies stratified by year of pregnancy beginning (orange line. y-axis on the right-hand side) and distribution of pregnancies according to time window with last TNFi dispensation (bars. y-axis on the left-hand side)

Overall, 1,096 pregnancies with 1,113 live-born children fulfilled the inclusion criteria. Figure 1 shows the number of included pregnancies by year of pregnancy onset. The number steadily increased during the study period. For example, it was nine in 2005, 60 in 2011 and 209 in 2017. Figure 1 also shows the distribution of pregnancies according to the last dispensing of TNFi medication in the period “one year before pregnancy to delivery”. The proportion with a last dispensing in the 2nd or 3rd trimester increased during the study period. For example, the proportion was 11.1% in 2005, 30.0% in 2011 and 41.6% in 2017. As shown in Supplementary Fig. 1, these patterns were rather similar when restricting the analysis to TNFi with a high placental transfer. Throughout the study period, higher proportions with TNFi dispensations in the 2nd and 3rd trimester were observed for pregnancies with maternal diagnosis of inflammatory bowel disease as compared to pregnancies with maternal diagnosis of inflammatory rheumatic disease (see Supplementary Figures S1.3 and S1.4).

**Fig. 2:**
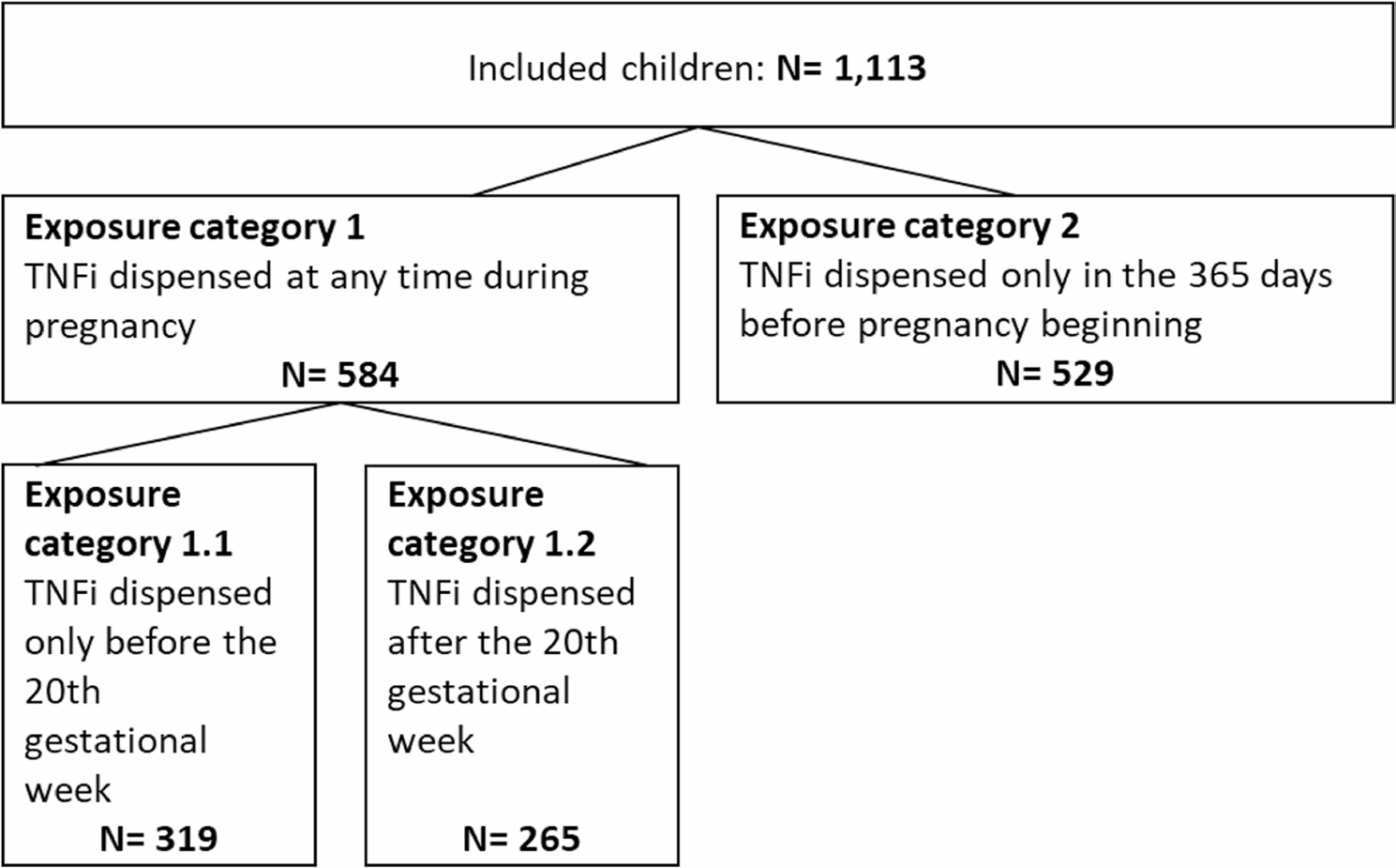
Number of included children overall and stratified by exposure category

Among live-born children (*n* = 1,113), there was a maternal exposure to TNFi only in the 365 days prior to pregnancy onset in *n* = 529 (47.5%) (i.e., no dispensing during pregnancy). In 319 children (28.7%), there was a dispensing of TNFi between pregnancy onset and the 20th gestational week but not later during pregnancy. In 265 children (23.8%), TNFi were dispensed for maternal treatment after 20th week of gestation (Fig. 2).

Table 1 describes these three subgroups with respect to maternal characteristics and pregnancy-related characteristics. The mean age of the mother was between 31 and 32 years in all three subgroups. Among children exposed only in the 365 days before pregnancy, 68% of mothers had a diagnosis code of inflammatory rheumatic disease and 32% of inflammatory bowel disease. Among children exposed after the 20th gestational week, 37% of mothers had a diagnosis code of inflammatory rheumatic disease and 65% of inflammatory bowel disease. The proportion of children born before the 37th gestational week ranged between 11% and 13% across subgroups. The proportion born by caesarean section was 42% in children exposed only before pregnancy and between 49% and 50% in children exposed during pregnancy.

**Table 1:** Description of children exposed to TNFi regarding maternal characteristics and pregnancy-related characteristics overall and stratified by exposure category

	Exposure category 1		Exposure category 2	Overall
	Exposure category 1.1	Exposure category 1.2		
	TNFi only before the 20th gestational week	TNFi after the 20th gestational week	TNFi only in the 365 days before pregnancy	
	*N* = 319	*N* = 265	*N* = 529	*N* = 1113
Characteristics of the mother				
Age at the beginning of pregnancy				
Mean (SD)	31.8 (4.6)	31.8 (4.3)	31.4 (4.5)	31.6 (4.5)
Median (Q1-Q3)	32 (29; 35)	32 (29; 34)	31 (28; 35)	32 (29; 35)
Diagnoses codes before pregnancy*				
Inflammatory rheumatic disease	155 (48.6%)	98 (37.0%)	359 (67.9%)	612 (55.0%)
Rheumatoid arthritis	107 (33.5%)	67 (25.3%)	250 (47.3%)	424 (38.1%)
Ankylosing spondylitis	54 (16.9%)	36 (13.6%)	124 (23.4%)	214 (19.2%)
Juvenile idiopathic arthritis	25 (7.8%)	18 (6.8%)	53 (10.0%)	96 (8.6%)
Psoriasis arthritis	30 (9.4%)	8 (3.0%)	78 (14.7%)	116 (10.4%)
Inflammatory bowel disease	163 (51.1%)	171 (64.5%)	168 (31.8%)	502 (45.1%)
Crohn’s disease	130 (40.8%)	134 (50.6%)	136 (25.7%)	400 (35.9%)
Ulcerative colitis	64 (20.1%)	63 (23.8%)	61 (11.5%)	188 (16.9%)
Other diseases	59 (18.5%)	18 (6.8%)	89 (16.8%)	166 (14.9%)
Acne inversa	1 (0.3%)	0 (0.0%)	0 (0.0%)	1 (0.1%)
Plaque psoriasis	48 (15.0%)	15 (5.7%)	68 (12.9%)	131 (11.8%)
Uveitis	11 (3.4%)	3 (1.1%)	21 (4.0%)	35 (3.1%)
Pregnancy-related characteristics				
Number of pregnancies	317 (100%)	260 (100%)	519 (100%)	1,096 (100%)
Multiple births per pregnancy				
Two live-borns	2 (0.6%)	5 (1.9%)	8 (1.5%)	15 (1.4%)
More than two live-borns	0 (0.0%)	0 (0.0%)	1 (0.2%)	1 (0.1%)
Pregnancy duration				
Mean (standard deviation)	272.1 (15.9)	273.5 (13.2)	271.9 (16.3)	272.4 (15.5)
Median (interquartile range)	275 (266; 283)	275 (269; 283)	276 (267; 282)	276 (267; 282)
≥ 37 weeks	281 (88.1%)	235 (88.7%)	458 (86.6%)	974 (87.5%)
≥ 34 - <37 weeks	30 (9.4%)	25 (9.4%)	50 (9.5%)	105 (9.4%)
≥ 31 - <34 weeks	3 (0.9%)	5 (1.9%)	13 (2.5%)	21 (1.9%)
≥ 28 - <31 weeks	3 (0.9%)	0 (0.0%)	6 (1.1%)	9 (0.8%)
< 28 weeks	2 (0.6%)	0 (0.0%)	2 (0.4%)	4 (0.4%)
Caesarian section	159 (49.8%)	130 (49.1%)	221 (41.8%)	510 (45.8%)

* Percentages do not add up to 100% given that codes for different diseases may have been recorded in one woman

**Fig. 3:**
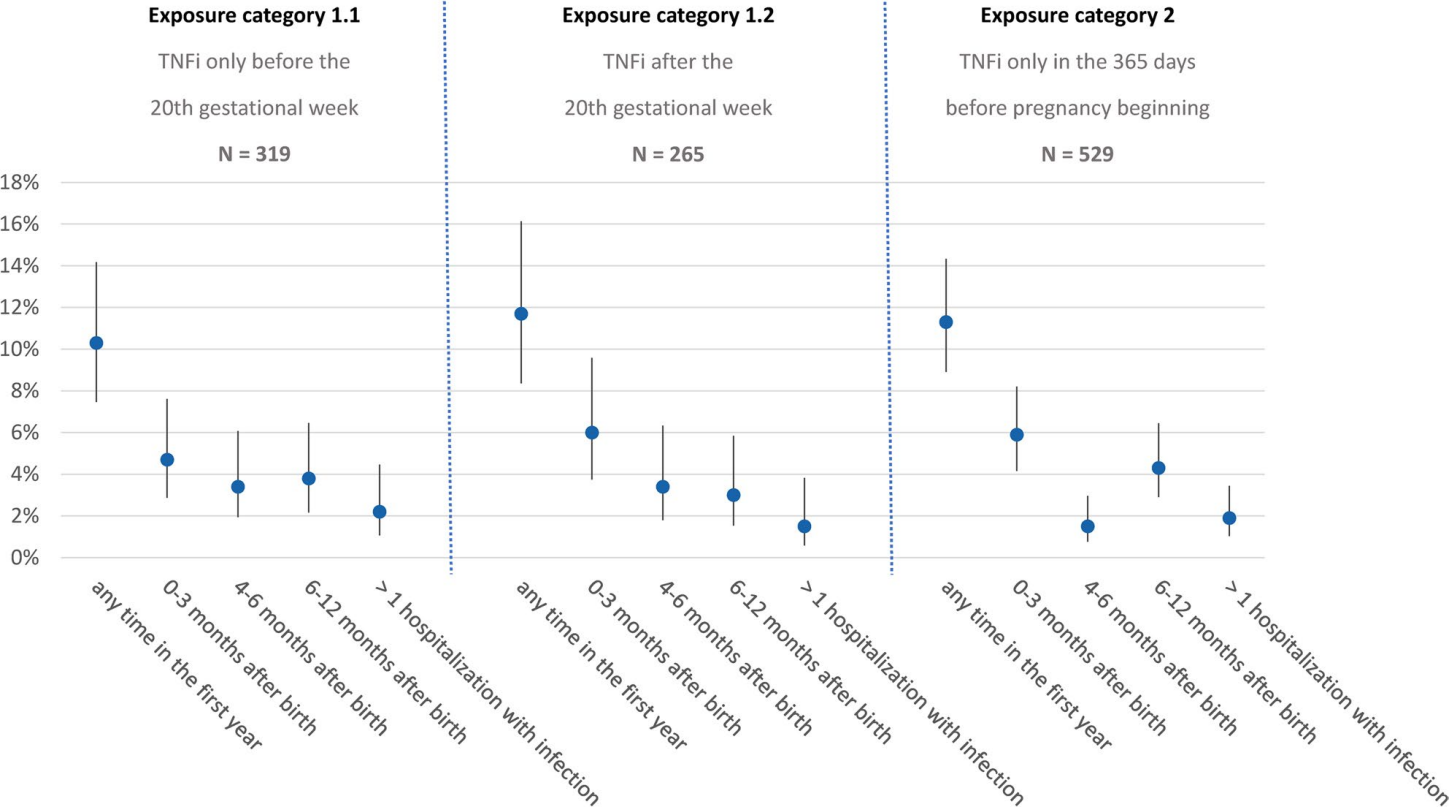
Proportion of children hospitalized with an infection in the first year after birth by exposure category - TNFi (overall)

Figure 3 shows the proportion of children hospitalized due to an infection in the first year of life for each of the three subgroups. In children exposed only in the 365 days before pregnancy, the proportion was 11.3% (95% confidence interval: 8.9%−14.3%). In those exposed until the 20th gestational week, the proportion was 10.3% (95% confidence interval: 7.5%−14.2%) and in those exposed after the 20th gestational week, the proportion was 11.7% (95% confidence interval: 8.4%−16.1%). In all subgroups, hospitalization due to an infection was more common in the first quarter of the first year compared to the second, third or fourth quarter. Also, the proportion of children with more than one hospitalization due to an infection in the first year of life was similar across subgroups (Fig. 3). The pattern did not change when we restricted the analyses to children exposed to TNFi with high placental transfer (Supplementary Figure S2). Supplementary Figure S3 shows the proportion of children hospitalized any time in the first year of live according to exposure category for different kinds of infection.

## Discussion

In this population-based study, we included data on 1,113 children born between 2006 and 2018 from mothers who had at least one dispensing of TNFi in the 365 days before or during pregnancy. The total number of children included per year and the number of children exposed during pregnancy increased during the study period. This also applied to the proportion of children exposed during the 2nd or 3rd trimester. The proportion of children hospitalized with a severe infection in the first year of life was similar among those born to mothers with a TNFi dispensing only before pregnancy and those who received a TNFi dispensing in the first or second half of pregnancy. The results regarding prescribing patterns and infections hardly changed when we focused on TNFi with high placental transfer.

Our finding that severe infections were not more common among children exposed in the second half of pregnancy as compared to children exposed earlier or unexposed during pregnancy, which is based on merely descriptive analyses, is consistent with results from many other studies. Gubatan et al. summarized nine studies on this research question published up to June 2020 on pregnant women with inflammatory bowel disease. In this meta-analysis, which included 8,013 women who gave birth to 8,490 infants, the use of TNFi during pregnancy did not show a statistically significant association with infection-related hospitalization in infants (OR 1.33, 95% CI 0.95–1.86) [17]. Also, more recent studies from the Netherlands, the United States and France did not show a higher risk of (serious) infections during the first year of life or later among children exposed *in utero* to TNFi compared to unexposed children [18–20]. The population-based study using national health data from France only observed an increased risk of serious infections in children exposed to both thiopurine and TNFi (adjusted HR 1.36; 95% CI, 1.04–1.79) [20] A further study based on data from Denmark, Finland and Sweden investigating TNFi exposure during pregnancy prescribed for any indication reported an increased risk of pediatric infections in the first year of life compared to the general population. Medical birth registers, patient registers and prescribed drug registers provided the data basis. As the study found an increased risk regardless of treatment in the 3rd trimester, the authors stated as one explanation that this may also be due to residual confounding by disease severity and healthcare-seeking behavior [14]. A similar study using data from Danish health registries also found an increased risk of any infections compared to controls from the general population [22]. Generally, when comparing studies on this research topic it is important to bear in mind that they are heterogeneous regarding the type of analysis (merely descriptive vs. causal analysis), the endpoint (e.g., any infections or serious infections), the control group (e.g., children from mothers with the same disease or from the general population) and (in the case of causal analysis) the possibilities to consider confounding. Assuming that disease severity was an important unmeasured confounder in these studies (exposure to TNFi more often in those with severe disease and severe disease as risk factor regarding child health), it is important to note that this would lead to overestimating rather than underestimating the risk of TNFi during pregnancy. As regards the overall proportion of children exposed to TNFi in utero and hospitalized due to severe infections, it likely depends on the health system, but the proportion observed in our study (~ 11%) was similar to proportions reported by studies from other European countries such as the Netherlands and Belgium (10%) [18, 21].

With respect to prescribing patterns, our study suggests that physicians in Germany are becoming more confident in prescribing TNFi during pregnancy. Obviously, this also applies to prescribing them in the second half of pregnancy and to TNFis with high placental transfer. This may reflect the fact that consensus papers published during the study period were already rather vague with respect to discontinuation and emphasized uncertainty whether TNFi actually increase the risk of severe infections [8, 12]. It may also reflect that after publication of these consensus papers, several studies with rather reassuring results were published, as mentioned above.

There are not many studies to which we can compare our findings regarding prescribing patterns in Germany. A recent study by Blaschke et al. used claims data from 2013 to 2017 and reported on overall treatment patterns and healthcare resource utilization in pregnant women with inflammatory rheumatic disease or psoriatic disease, i.e., the information provided specifically on TNFi was limited and the study was restricted by indication. In this study, TNFi were prescribed to 13.1% of women with inflammatory rheumatic disease before pregnancy, which decreased to 7.7% of women during pregnancy course, i.e., ~ 60% of those using TNFi before pregnancy continued treatment during pregnancy [39]. In our analysis, the overall proportion of women continuing treatment during pregnancy was lower (45%) but our study also included data from earlier years. The results for more recent years described here are consistent with the proportion reported by Blaschke et al.

Our study has specific strengths and limitations. The database we used covers 20% of the German population, it is free of non-response and recall bias and reflects the real-world healthcare setting in Germany. We had information on TNFi use in both the inpatient and outpatient setting including the date of dispensation (outpatient) and the date of application (inpatient). As always in pharmacoepidemiological studies, there is some uncertainty regarding actual drug use but this uncertainty is lower for drugs administered as injections and for those used in chronic conditions, which both applies to TNFi. Regarding the classification of the time of the exposure we used the date of dispensation, prioritizing a high specificity over a high sensitivity (e.g., in a woman with an infusion in week 18, it is less certain whether there are high concentrations in week 21 as compared to a woman with an infusion in week 21). The occurrence of serious infections in infants was assessed based on hospital discharge diagnoses, which are known to have a high validity as they are checked for accuracy by the Health Insurance Medical Service. In addition, a validation study conducted for French claims data confirmed the validity for infections overall coded in the inpatient setting [40]. It is not clear whether this also applies to the distinction of the different types of infections, but the focus of our paper was not on subtypes of infections. There might also be serious infections treated in the outpatient setting but with claims data it is hardly possible to distinguish severe from less severe infections, i.e., it would lead to substantial misclassification. Comparing the frequency of severe infections in the first year of life between our study population (i.e., infants born from mothers using TNFi before or during pregnancy) with infants from the general population would also have been of interest, but our dataset did not contain such a comparison group.

Furthermore, we used an algorithm that estimates the onset of pregnancy based on the expected delivery date for the vast majority of pregnancies, which is expected to minimize misclassification of gestational age [30, 31]. As we included pregnancies ending between 2006 and 2018, we could also describe prescribing patterns over time. Even though our study included 1,113 children of whom 265 were exposed to TNFi in the second half of pregnancy it should be kept in mind that this sample size has limited power to detect small differences in the proportions of serious infections between groups. As mentioned above, it is also important to consider that our study was merely descriptive. Estimating causal effects of TNFi on the risk of serious infections would have required another design including the consideration of the different types of indications as separate groups as well as relevant confounders. The latter include maternal disease severity and activity, breastfeeding practices, healthcare-seeking behavior and socioeconomic status as well as other relevant drug exposures. Our descriptive study is helpful to assess the possibility of future causal analyses and the suitability of the available data for this purpose.

In conclusion, our analyses showed that among children whose mothers used TNFi in the year before or during pregnancy, about one fourth was exposed to TNFi in the second half of pregnancy. Among these children, the proportion hospitalized with a severe infection in the first year of life was similar to that from mothers exposed only before pregnancy.

## Supplementary Information

Supplementary Material 1.

## Data Availability

In accordance with German data protection regulations, access to the data of the German Pharmacoepidemiological Research Database may only be given to third parties within the realm of collaborations with BIPS and after signing an agreement for guest researchers. Furthermore, as we are not the owners of the data we are not legally entitled to grant access to the data or to store data elsewhere, e.g., in a repository. This also relates to any kind of analysis datasets extracted from GePaRD.

## Abbreviations

ADA: Adalimumab
ATC: Anatomical Therapeutic Chemical
CI: Confidence interval
CTZ: Certolizumab pegol
EBM: Einheitlicher Bewertungsmaßstab
ETA: Etanercept
GePaRD: German Pharmacoepidemiological Research Database
GOL: Golimumab
ICD-10-GM: International Statistical Classification of Diseases and Related Health Problems, version 10 in the German Modification
IFX: Infliximab
OPS: German coding system for operations and procedures (“Operationen- und Prozedurenschlüssel”)
TNFi: Tumor necrosis factor inhibitors
