# Supplementary material for: Treatment with tumor necrosis factor inhibitors during pregnancy and occurrence of severe infections among exposed infants in Germany
Source: BMC Pregnancy Childbirth. 2025 Dec 20;26:78. doi: 10.1186/s12884-025-08590-0 (PMC12831379; doi:10.1186/s12884-025-08590-0)
Supplement: Supplementary file 1 — Supplementary Material 1. [file 12884_2025_8590_MOESM1_ESM.docx]

**S Supplementary material**

**Supplementary Figure S1.1:** Number of included pregnancies with a dispensation of a TNFi with high placental transfer (IFX, ADA, GOL) stratified by year of pregnancy beginning (orange line. y-axis on the right-hand side) and distribution of pregnancies according to time window with last dispensation of IFX, ADA or GOL (bars. y-axis on the left-hand side)

**Supplementary Figure S1.2:** Number of included pregnancies with a dispensation of a TNFi with low placental transfer (CTZ, ETA) stratified by year of pregnancy beginning (orange line. y-axis on the right-hand side) and distribution of pregnancies according to time window with last dispensation of CTZ or ETA (bars. y-axis on the left-hand side)

**Supplementary Figure S1.3:** Number of included pregnancies with maternal diagnosis of inflammatory rheumatic disease stratified by year of pregnancy beginning (orange line. y-axis on the right-hand side) and distribution of pregnancies according to time window with last dispensation (bars. y-axis on the left-hand side)

**Supplementary Figure S1.4:** Number of included pregnancies with maternal diagnosis of inflammatory bowel disease stratified by year of pregnancy beginning (orange line. y-axis on the right-hand side) and distribution of pregnancies according to time window with last dispensation (bars. y-axis on the left-hand side)

**Exposure category 2**

TNFi only in the 365 days

before pregnancy beginning

**N = 529**

**Exposure category 1.1**

TNFi only before the

20th gestational week

**N = 319**

**Exposure category 1.2**

TNFi after the

20th gestational week

**N = 265**

**Supplementary Figure S2:** Proportion of children hospitalized with an infection in the first year after birth by exposure category - TNFi with high placental transfer (IFX, ADA, GOL)

**Supplementary Figure S3:** Proportion of children hospitalized with an infection in the first year after birth, stratified by kind of infection and by exposure category (Exposure category 1.1= TNFi only before the 20th gestational week, exposure category 1.2= TNFi after the 20th gestational week, exposure category 2= TNFi only in the 365 days before pregnancy beginning)

**Supplementary Table S1:** ATC/ OPS codes to identify TNF-alpha inhibitors of interest

| **TNFi** | **ATC code** | **OPS code(s)** |
| --- | --- | --- |
| Adalimumab | L04AB04 | 6-001.d-, 8-012.t- |
| Certolizumab pegol | L04AB05 | 6-005.7 |
| Etanercept | L04AB01 | 6-002.b-, 8-013.b- |
| Golimumab | L04AB06 | 6-005.2 |
| Infliximab | L04AB02 | 6-001.e-, 8-012.7- |

**Supplementary Table S2:** ICD-10-GM codes used to identify relevant diseases in mothers

| **Disease** | **ICD code(s)** |
| --- | --- |
| *Inflammatory rheumatic disease (IRD)* | |
| Rheumatoid arthritis | M05-, M06- |
| Ankylosing spondylitis | M081-, M450- |
| Juvenile idiopathic arthritis | M08-, M090- |
| Psoriatic arthritis | M07-, M09-, L405 |
| *Inflammatory bowel disease (IBD)* | |
| Crohn’s disease | K50-, M074-, M091- |
| Ulcerative colitis | K51-, M075-, M092- |
| *Other diseases (OD)* | |
| Plaque psoriasis | L400, L401, L402, L403, L404, L407, L4070, L408, L409 |
| Acne inversa | L732 |
| Uveitis | H20-, H30- |

**Supplementary Table S3:** ICD-10-GM codes to identify severe infections (cf. Bröms et al., 2019)

| **Upper respiratory** |  |  |
| --- | --- | --- |
|  | Abscessus peritonsillaris | J36 |
|  | Ear | H601-H603, H65-H67, H70 |
|  | Laryngitis | J04–J05 |
|  | Nasopharyngitis | J00 |
|  | Pharyngitis | J02, J391-J392 |
|  | Sinusitis, ethmoiditis | J01 |
|  | Tonsillitis | J03 |
|  | Other | J06 |
| **Lower respiratory** |  |  |
|  | Pneumonia | A70, J13-J18 |
|  | Other, including whooping cough | A37, J20-J22, J85-J86 |
| **Gastrointestinal** |  |  |
|  | Intestinal infections | A00-A09 |
|  |  | K040, K044, K047, K050, K052, K113, K122, K35-K37, K61 |
| **Viral** |  |  |
|  | Influenza and respiratory viruses | J09-J12 |
|  | Encephalitis, poliomyelitis, meningitis | A80-A89, G02, G05 |
|  | Other viral | A60-A63, A90-A99, B00-B02, B04-B06, B08-B09, B15-B27, B30, B33-B34, B97 |
|  | Congenital viral diseases | P35 |
| **Other** |  |  |
|  | Skin, erysipelas, lymphadenitis | A46, H600, L00-L08 |
|  | Bacterial | A20-A36, A38-A39, A43-A44, A48-A54, A65-A79, B95-B98 |
|  | Bacterial sepsis, meningitis and other infections of perinatal period | A40-A41, G00-G01, G03-G04, G06-G09, P36-P39 |
|  | Tuberculosis | A15-A19 |
|  | Mycoses | A42, B35-B49 |
|  | Urinary tract | N136, N30.0, N39.0 |
|  | Circulatory system | I00-I01, I301, I33, I400, I410, I412, I430, I520-I521 |
|  | Musculoskeletal | M00-M01, M462-M465, M630, M632 M726, M86 |
|  | Tropical | B50-B94 |
